# Regional Differences in Susceptibility to Hypoxic-Ischemic Injury in the Preterm Brain: Exploring the Spectrum from White Matter Loss to Selective Grey Matter Injury in a Rat Model

**DOI:** 10.1155/2012/725184

**Published:** 2012-03-15

**Authors:** D. B. Selip, L. L. Jantzie, M. Chang, M. C. Jackson, E. C. Fitzgerald, G. Boll, A. Murphy, F. E. Jensen

**Affiliations:** ^1^Newborn Medicine, Children's Hospital Boston, and Harvard Medical School, 300 Longwood Avenue, Boston, MA 02115, USA; ^2^Department of Neurology, Children's Hospital Boston, and Harvard Medical School, 300 Longwood Avenue, Boston, MA 02115, USA; ^3^Program in Neurobiology, Harvard Medical School, Boston, MA 02115, USA

## Abstract

Models of premature brain injury have largely focused on the white matter injury thought to underlie periventricular leukomalacia (PVL). However, with increased survival of very low birth weight infants, injury patterns involving grey matter are now recognized. We aimed to determine how grey matter lesions relate to hypoxic-ischemic- (HI) mediated white matter injury by modifying our rat model of PVL. Following HI, microglial infiltration, astrocytosis, and neuronal and axonal degeneration increased in a region-specific manner dependent on the severity of myelin loss in pericallosal white matter. The spectrum of injury ranged from mild, where diffuse white matter abnormalities were dominant and were associated with mild axonal injury and local microglial activation, to severe HI injury characterized by focal MBP loss, widespread neuronal degeneration, axonal damage, and gliosis throughout the neocortex, caudate putamen, and thalamus. In sum, selective regional white matter loss occurs in the preterm rat concomitantly with a clinically relevant spectrum of grey matter injury. These data demonstrate an interspecies similarity of brain injury patterns and further substantiates the reliable use of this model for the study of preterm brain injury.

## 1. Introduction

Preterm deliveries make up more than 500,000, or approximately 12.5 percent, of all infant births in the United States [1]. Although technological advances in neonatal care have dramatically improved the survival rates for the smallest and youngest infants, such advances have yet to fully protect the developing brain from injury and prevent the neurological morbidities associated with prematurity. Of those infants born less than 32 weeks gestational age and weighing less than 1500 g (very low birth weight, VLBW), approximately 10% have motor deficits and up to 60% have neurocognitive disabilities and/or behavioral issues [2, 3]. The most common predisposing factors to premature brain injury are hypoxia-ischemia (HI) and/or sepsis [4–7]. However, all premature newborns are at risk for brain injury and a specific ischemic episode is not required [8]. Specifically, *in utero* HI events (placental insufficiency, chronic fetal-to-maternal hemorrhage, stroke, infection, and inflammation), perinatal events (placental abruption, respiratory failure), and neonatal disorders (chronic lung disease, congenital cardiac abnormalities) are associated with acquired brain injuries that lead to cerebral palsy, intellectual disability, epilepsy, visual and hearing impairment, and issues with school readiness [8–12]. Further, the risk of brain injury and abnormal brain development in the premature newborn can be altered by systemic illness and by critical care therapies [10, 13]. In addition to the individual familial burdens of caring for infants and children with these disabilities, the socioeconomic impact of such care in the United States is estimated to cost in excess of $26.2 billion a year [1].

The neuropathology of premature brain injury is diverse and comprised of multiple lesions. The most commonly observed is periventricular leukomalacia (PVL), and it occurs in greater than 50% of VLBW infants [14, 15]. Historically, it was believed that white matter was exclusively injured following HI in preterm infants, as macroscopic focal necrotic lesions and cysts were easily identifiable on standard, acute cranial ultrasonography [16]. Over the past 10–15 years, cystic PVL has declined in incidence and currently occurs in less than 5% of VLBW infants [15]. However, the increasing application of MRI to the clinical assessment of brain injury in the preterm newborn has now revealed that diffuse noncystic white matter injury is the dominant pattern of white matter injury [13, 17], accounting for more than 90% of PVL and occurring in up to half of premature VLBW newborns [13, 15, 18, 19]. Diffuse PVL is a cell-specific lesion consisting of acute loss of early differentiating/premyelinating oligodendrocytes (preOLs) with accompanying astrogliosis and microgliosis, followed by a deficit in mature myelin producing OLs and subsequent cerebral hypomyelination [16]. The routine use of more advanced MRI methodologies also indicate that cerebral white matter abnormalities are accompanied by injury to grey matter structures in the cerebrum, diencephalon, brain stem, and cerebellum in preterm infants [8, 14]. Increasingly, the term “encephalopathy of prematurity” is used to describe PVL and the associated neuronal/axonal abnormalities and is believed to accurately represent the complex brain injury observed in this patient population [14, 20, 21]. However, it remains undefined how severity of insult relates to the pattern of injury observed.

Many studies conducted in animals and humans have investigated the etiology and pathophysiology of preterm brain injury and its developmental sequelae [22–28]. Following HI, with or without underlying infection, an intricate cascade of cellular injury comprised of excitotoxic, oxidative, and inflammatory events converge to produce cell death in an immature brain that is temporally and developmentally vulnerable [14, 16]. Although cerebral ischemia and systemic infection/inflammation are the two major upstream pathogenic mechanisms, preOLs are intrinsically vulnerable in the preterm brain and are immensely susceptible to excitotoxicity, microglial activation, and free radical attack [16, 28–31]. We have studied the pathophysiology of HI-injury *in vivo*, using a rat model of unilateral carotid artery ligation (UCL) followed by hypoxia [25, 31, 32]. Many variations and modifications to this model have been made over the years allowing for the study of preterm and term equivalent brain injury. While there is considerable data from human pathological studies of encephalopathy of prematurity [14], few rodent studies have addressed the relative susceptibilities of different brain regions exposed to HI injury at a preterm equivalent age. The goal of this study was to investigate the regional relationships, susceptibilities, and patterns of HI induced grey matter injury as it relates to a clearly defined spectrum of diffuse white matter injury in the preterm rodent brain. It was hypothesized that mild HI would result in white matter injury alone, and that an increase in the severity of HI-induced white injury would result in an increase in the severity and the regional diversity of cortical and subcortical grey matter injury.

## 2. Materials and Methods

### 2.1. Varying Hypoxic-Ischemic (HI) Injury with Carotid Artery Ligation and Hypoxia

All procedures were approved and in accordance with guidelines set forth by the Animal Care and Use Committee of Children's Hospital Boston (Boston, MA, USA). To perform carotid artery ligation, male P6 Long-Evans rat pups were anesthetized with ether. A midline incision at the base of the neck was made, and the left common carotid artery was exposed, isolated from the sympathetic chain and vagus nerve, and permanently ligated using a microelectrocauterizer. One-to-two midline sutures were placed and the neck wound closed. After surgery and recovery from sedation, but prior to hypoxia, pups were allowed to reside with dam for 1-2 hr to ensure full recovery and appropriate hydration. To induce hypoxia, pups were placed in a sealed, global hypoxic environment held at 6% O_2_ balanced N_2_. Normothermia was maintained throughout hypoxia with the aid of thermal blankets. Core body temperature was monitored by rectal probe prior to and after surgery. Surgical times and weights of the animals at P6 and P9 were obtained (Table 1).

To create a spectrum of HI-induced brain injury, pups were randomized to one of five groups of hypoxia exposure duration. These groups included: exposure to 6% O_2_ for 1 hr 0 min (*n* = 10), 1 hr 5 min (*n* = 9), 1 hr 10 min (*n* = 8), 1 hr 15 min (*n* = 11), or 1 hr 20 min (*n* = 11). Litter-matched sham controls were neither subject to carotid ligation nor hypoxia (Table 1). Following hypoxia, pups were returned to their dams until euthanasia.

### 2.2. Histological and Immunohistochemical Analysis of Brain Injury

All pups (control and UCL/hypoxia) were euthanized by terminal pentobarbital anesthesia followed by intracardiac perfusion of PBS and 4% paraformaldehyde (PFA) at 72 hr (P9). Brains were then removed and postfixed in 4% PFA, at 4°C, followed by cryoprotection in 30% sucrose. Serial, 16 *μ*m, coronal sections were obtained via cryostat (Leica CM3050S) and collected from each animal at the level of the anterior hippocampus through to the posterior hippocampus. Hematoxylin and eosin (H&E) staining and Fluoro-Jade B (FJB) (Chemicon) staining were performed according to standard and manufacturer protocols. Immunohistochemistry was performed as previously published [29, 31, 33, 34] using the following primary antibodies: mouse monoclonal antibodies to myelin basic protein (MBP/SMI-99, 1 : 1000, Covance), CD68 (1 : 100, Serotec), and glial fibrillary acidic protein (GFAP/SMI-22, 1 : 1000, Covance); rabbit polyclonal antibody to fractin (1 : 1000, Chemicon). Briefly, sections were blocked with 5% normal goat serum and then incubated overnight at 4°C with the appropriate primary antibody. The following day, a species appropriate secondary antibody (goat anti-mouse Alexa Fluor 488 or 568, Invitrogen) was applied to the slides for 1 hr at room temperature. Slides were then rinsed and cover-slipped with antifade medium (Fluoromount-G; Southern Biotechnology). Images were obtained on a Zeiss Axioscope, using a Spot Digital Camera and Advanced 4.5 software (Diagnostic Instruments).

### 2.3. Scoring and Image Analysis

An observer blinded to every aspect of the experimental protocol performed all scoring and image analyses. H&E sections were evaluated by light microscopy for cell loss, pyknotic nuclei, dense areas of eosinophilia, and macrocyst formation in periventricular white matter (WM) and overlying temporal-parietal cortex. Using our previously published semiquantitative scoring system [31], loss of WM was measured by Image J quantification of 2.4 mm^2^ field of MBP in periventricular WM at the level of the middorsal hippocampus, 2.8–3.1 mm from bregma, 2.6–3.0 mm lateral to midline, and anatomically similar cross-sections [35]. The total area of MBP staining ipsilateral to UCL/hypoxia was compared to total area of MBP in the hemisphere contralateral to carotid ligation to determine percent WM change in the HI animals and percent WM in sham controls. Pups were then stratified into groups based on the percent of WM loss in the periventricular region ipsilateral to carotid ligation as compared to the contralateral hemisphere. Analysis groups were assigned as follows. Grade 0: *no discernable MBP loss *(0% reduction in MBP ipsilateral to carotid ligation as compared to contralateral WM), Grade 1: *mild MBP loss* (1–37% reduction in MBP ipsilateral to carotid ligation compared to contralateral WM), Grade 2: *moderate MBP loss* (38–69% reduction in WM ipsilateral carotid ligation compared to contralateral WM), and Grade 3: *severe MBP loss* (70–100% reduction in MBP ipsilateral to carotid ligation compared to contralateral WM) [31]. All sham control animals were scored using the same methodology.

Gliosis and evidence of neuroinflammation were defined by concurrent reactive astrocytosis and activated microglia and were identified by immunostaining for GFAP and CD68, respectively. Neuronal degeneration and axonal injury were identified using FJB and fractin immunostaining, respectively. Regions evaluated were the periventricular WM and overlying temporal-parietal cortex, hippocampus, thalamus, internal capsule, and caudate putamen as per the Stereotactic coordinates listed above. For each stain/immunostain, scoring was evaluated on a 0–3 point scale based on the density of FJB/immunopositive cells, where 0: no GFAP, CD68, fractin or FJB-positive cells; 1: diffuse areas of mild staining/immunoreactivity; 2: moderate staining/immunoreactivity occurring in dense, focal or columnar patches; 3: widespread severe staining/immunoreactivity distributed throughout the entire brain region (Figures 2 and 3).

### 2.4. Statistical Analysis

Data is expressed as mean ± standard error of the mean (SEM). Normally distributed data differences between two groups were compared using Student's *t*-test. Multiple groups were compared using one-way ANOVA with the Bonferroni multiple-comparison *post hoc* test. Nonparametric datasets were compared using the Mann-Whitney rank sum test. A *P* value of ≤0.05 was considered statistically significant. Data were analyzed with SigmaStat 3.11 software (Systat Software 2004).

## 3. Results

### 3.1. Graduated Increase of Periventricular White Matter Loss with Lengthening Periods of Hypoxia after Carotid Ligation

**Figure 1:**
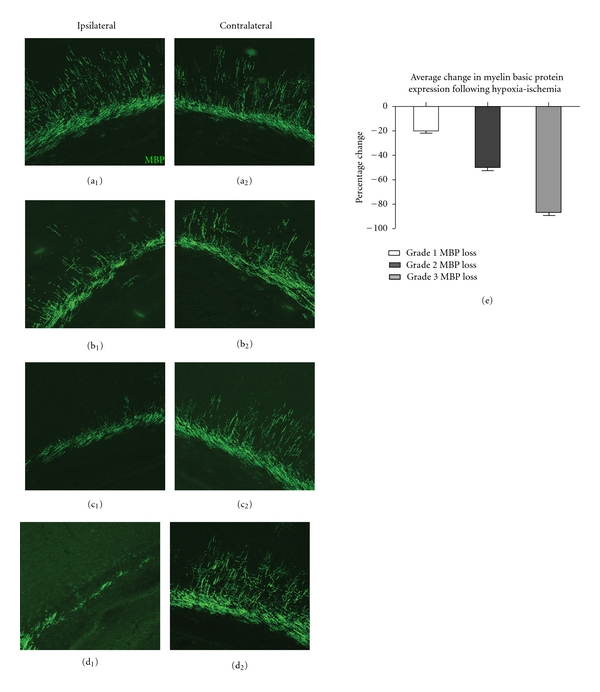
Periventricular white matter loss following hypoxia-ischemia (HI) in postnatal day 6 (P6) rats. Seventy-two hours following HI at P6, myelin basic protein (MBP) is significantly depleted in the hemisphere ipsilateral to carotid ligation. Representative photomicrographs show MBP in hemispheres both ipsilateral (a_1_–d_1_) and contralateral (a_2_–d_2_) to carotid ligation in animals with Grade 0 MBP loss (no discernable white matter injury, a_1_-a_2_), Grade 1 MBP loss (mild white matter injury, b_1_-b_2_), Grade 2 MBP loss (moderate white matter injury, c_1_-c_2_), and Grade 3 MBP loss (severe white matter injury, d_1_-d_2_). Histogram shows average percent change in MBP for each group (e).

A total of 49 HI rat pups and 10 litter matched sham control pups (no surgery, no hypoxia) were evaluated in this study. There were no statistically significant differences in weight at P6 or P9, weight gain/growth over the 72 hr evaluation period, core body temperature at start or end of surgery, overall core body temperature decrease during surgery, and time for UCL surgical procedure between rats that had different durations of hypoxia (1 hr 0 min, 1 hr 5 min, 1 hr 10 min, 1 hr 15 min, and 1 hr 20 min) after UCL at P6 (Tables 1 and 2). However, the different durations of hypoxia resulted in a spectrum of WM loss from undetectable to mild, moderate, and severe as evidenced by a graduated reduction in MBP immunoreactivity at P9 (Figure 1). Coronal sections from all HI animals (HI) were evaluated and then classified in myelination groupings as no MBP loss (Grade 0; *n* = 16), mild MBP loss (Grade 1; *n* = 13), moderate MBP loss (Grade 2; *n* = 9), and severe MBP loss (Grade 3; *n* = 11) based on percent WM reduction (Table 2). Animals with mild MBP loss had a mean reduction in MBP ipsilateral to carotid ligation of 20.21 ± 2.09%, *P* < 0.001 (Figure 1(b)). Animals with moderate MBP loss had a mean reduction ipsilateral to carotid ligation of 50.11 ± 2.67%, *P* < 0.001 (Figure 1(c)), and animals with severe MBP loss animals had a mean reduction ipsilateral to carotid ligation of 86.02 ± 3.24%, *P* < 0.001 (Figure 1(d)).

### 3.2. Relationship of Inflammation and Gliosis to MBP Loss

**Figure 2:**
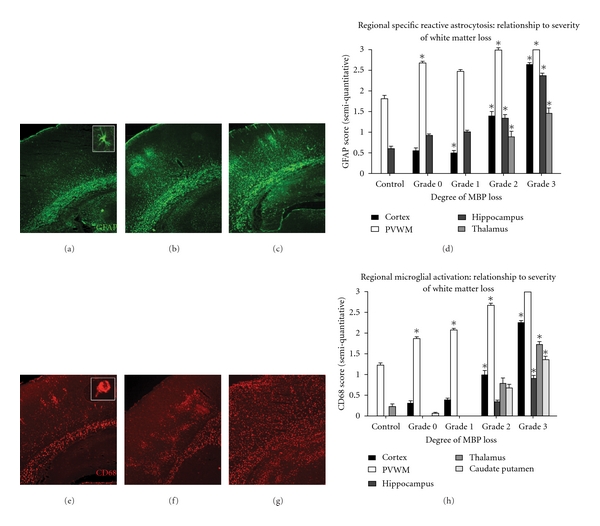
Astrogliosis and microgliosis in relation to severity of white matter injury in hypoxic-ischemic (HI) neonatal rats. Seventy-two hours following HI at postnatal day 6 (P6), numerous reactive astrocytes (a–d) and activated microglia (e–h) are present throughout the brains of neonatal rats with significant white matter injury. Representative photomicrographs depict GFAP-positive astrocytes and mild (a), moderate (b), and severe (c) astrogliosis following HI. Histogram in (d) displays the region-specific pattern and degree of reactive astrocytosis as a function of MBP loss. Lower panels depict CD68-positive microglia and mild (e), moderate (f), and severe (g) microgliosis following HI. Histogram in (h) shows the region-specific pattern and degree of microglial activation as a function of white matter injury severity. Magnification 40x.

Next, microglial activation was evaluated as a function of MBP loss (Figures 2(e)–2(h)). Of note, in uninjured sham controls activated microglia and reactive astrocytes were only observed in the white matter, hippocampus, and thalamus. All HI rat pups, including those with Grade 0 MBP loss, had significantly increased numbers of activated microglia within white matter as evidenced by increases in CD68 immunoreactivity compared to controls (mean score 1.88 ± 0.13 versus 1.22 ± 0.15, *P* < 0.01, Figure 2(h)). With increasing WM loss, there were further significant increases in the density of CD68 immunoreactivity within the white matter (mean score Grade 1: 2.08 ± 0.14; mean score Grade 2: 2.67 ± 0.17; mean score Grade 3: 3.0 ± 0.0, *P* < 0.01 for all, Figure 2(h)). In both Grade 1 and Grade 2 MBP loss groups, activated microglia were not only observed in WM but were present in a discrete, patchy columnar pattern in the overlying cortex (Figures 2(e)-2(f)). Accordingly, in brains displaying Grade 2 MBP loss, the density of activated microglia in the cortex was significantly increased compared to control (mean score 1.00 ± 0.29 versus 0.31 ± 0.18, *P* = 0.001). In brains exhibiting Grade 3 MBP loss, activated microglia were significantly more numerous and the columnar pattern to their distribution in the cortex was lost (mean score 2.22 ± 0.22). Specifically, the distribution of the CD68 positive cells was no longer detected in isolated focal patches and was increasingly widespread throughout the brain including the hippocampus (mean score 0.9 ± 0.25, *P* = 0.007), thalamus (mean score 1.72 ± 0.27, *P* < 0.001), and caudate putamen (mean score 1.36 ± 0.28, *P* < 0.001). 

Regional patterns and severity of reactive astrocytosis, as evidenced by significant increases in GFAP immunoreactivity in HI pups compared to controls, were similar to the patterns of activated microglia described above (Figures 2(a)–2(d)). Specifically, HI rat pups with Grade 0 MBP loss had a significant increase in WM reactive astrocytosis compared to controls (2.67 ± 0.19 versus 1.80 ± 0.29, *P* = 0.04). In contrast to pups with Grade 0 MBP loss that had numerous reactive astrocytes in the WM but not the cortex, those pups with Grade 1 MBP loss had a statistically significant increase in cortical reactive astrocytosis (mean score 0.5 ± 0.17, *P* = 0.03). In addition, as WM became increasingly injured in the pups with Grades 2 and 3 MBP loss, GFAP immunoreactivity similarly became increasingly dense in the WM (mean score Grade 2: 2.88 ± 0.11; mean score Grade 3: 3.00 ± 0.00) and extended throughout other areas of the brain including the hippocampus, and thalamus (*P* < 0.05 for all regions, Figure 2(d)).

### 3.3. Regional Predilection and Severity of Cortical and Subcortical Neuronal Degeneration and Axonal Injury as a Function of Periventricular White Matter Injury

**Figure 3:**
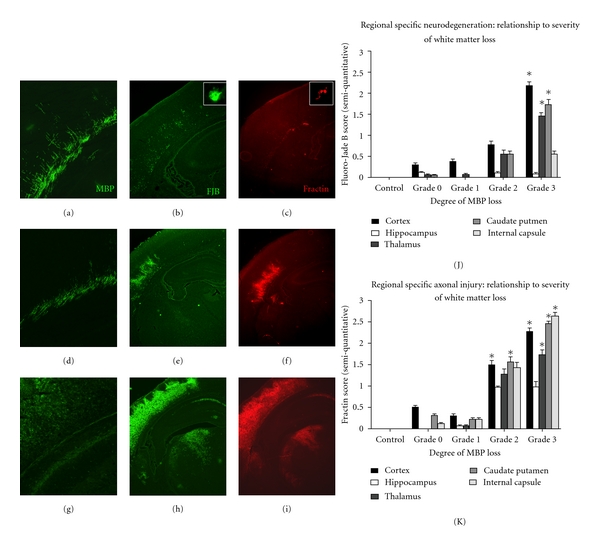
Significant grey matter injury accompanies myelin basic protein (MBP) loss in postnatal day 6 (P6) neonatal rats. Grey matter injury, as determined by neuronal and axonal degeneration, increases in hypoxic-ischemic neonatal rats with the severity of white matter injury. Representative photomicrographs show Grade 1 MBP loss (a), with mild cortical neuronal degeneration (FJB staining, (b)) and axonal injury (fractin immunoreactivity, (c)). With Grade 2 MBP loss (d), the density and distribution of FJB-positive neuronal cell bodies (e) and fractin-positive axons (f) increase in the cortex overlying the periventricular white matter. With Grade 3 MBP loss (g), the density of FJB-positive neuronal cell bodies (h) and fractin-positive spheroids (i) are further increased and encompasses the majority of the cortical mantle, as well as the hippocampus, thalamus, caudate putamen, and internal capsule. Histograms display region-specific neurodegeneration (j) and axonal injury (k) in relation to the severity of white matter loss. Magnification 100x for MBP and 25x for FJB and fractin.

Neuronal degeneration occurred more often and with greater severity as WM loss increased (Figure 3). In brains with Grade 3 MBP loss, FJB degenerating cells were significantly increased in the cortex overlying the white matter (mean score 2.18 ± 0.30, *P* = 0.001), thalamus (mean score 1.45 ± 0.34, *P* = 0.018), and caudate putamen (mean score 1.72 ± 0.38, *P* = 0.018).

In addition to assessing neuronal cell body degeneration, axonal injury was assessed with immunostaining for fractin (Figures 3(c), 3(f), 3(i), 3(k)). Unlike FJB, fractin immunoreactivity was present in brains with the mildest white matter injury and regional axonal injury was evident prior to the appearance of FJB-positive cells (Figures 3(j) and 3(k)). Despite this, no statistically significant differences in fractin protein expression was observed in Grade 0 and Grade 1 MBP loss brains, although there was a trend for an increase in these mildly injured brains (Figure 3(k)). In contrast, HI pups with moderate and severe WM loss had statistically significant increases in fractin immunoreactivity (Figures 3(f), and 3(i)). HI animals with Grade 2 MBP loss were observed to have moderate axonal injury in the cortex (mean score 1.51 ± 0.26, *P* = 0.015) and caudate putamen (mean score 1.55 ± 0.39, *P* = 0.043) compared to controls. Axonal injury was greatest in brains with Grade 3 MBP loss, with significant increases in fractin immunoreactivity in the cortex (mean score 2.27 ± 0.27, *P* < 0.001), thalamus (mean score 1.72 ± 0.41, *P* = 0.005), caudate putamen (mean 2.45 ± 0.21, *P* < 0.001), and internal capsule (mean score 2.64 ± 0.28, *P* < 0.001) (Figure 3(k)).

## 4. Discussion

Clinically, neuroimaging and postmortem analyses have shown that preterm brain injury involves both white and grey matter injury [8, 20, 36–38], and it has been difficult to generate a clinically relevant spectrum of HI pathology in neonatal rodents that closely resembles that observed in humans [9]. Although rodent models are inherently limited due to simplicity of brain structure relative to the human, the data presented here suggest that there is a spatiotemporal order of appearance of MBP loss, microglial and astrocytic infiltration, and neuronal somatic and axonal injury following HI. Specifically, we show that HI-mediated white matter injury, even when resulting in mild reductions of MBP, occurs in preterm equivalent rats concomitantly with significant white matter microglial activation and reactive astrocytosis. However, as the degree of periventricular/pericallosal white matter loss increases, the severity and frequency of cortical and subcortical grey matter injury also increase, with a widespread distribution of FJB-positive cells, fractin immunoreactivity, reactive astrocytes and activated microglia, and a regional predilection for the temporal-parietal cortex, internal capsule, caudate putamen, and thalamus. Further, as evidenced by the trend towards increased fractin-positive cells in the brains of animals with no discernable MBP loss, it is possible that mild grey matter injury may be present in this animal model even when periventricular WM loss is not. These findings are similar to injury patterns and susceptibilities noted in prior human preterm brain injury studies.

Recent experimental evidence, in combination with advanced imaging in the newborn, has led to “a blurring of the grey-white (term-preterm) dichotomy” [8]. It is now common to recognize white matter injury in the term baby and appreciate injury to grey matter structures, such as the thalamus and cerebellum, in the preterm brain [8]. In this investigation, we found that MBP loss appears to be the most sensitive measure of injury, and severity of MBP loss can be used as a benchmark in order to stage other pathophysiological responses such as microglial and astrocytic reactivity and neuronal injury. Interestingly, the damage to grey matter in the preterm rodent occurs in cortical and subcortical structures with similar pattern and distribution to that observed in the human preterm infant [14, 24, 39]. Importantly, these data corroborate the most important cellular aspects of the encephalopathy of prematurity described in the human, and the neuropathology documenting that neuronal loss and/or gliosis is present in 13–30% of cases of noncystic PVL [22]. We show that the cortex, thalamus, internal capsule, and caudate putamen are injured with HI exposure at P6 and that the severity and presence of this injury evaluated at P9 closely correlates to an increasing spectrum of periventricular WM loss. This is especially relevant when it is considered that a Long-Evans rat brain at P6 and P9 is developmentally similar to a 30-week and 40-week gestational age human brain, respectively [33, 34].

Many sequences of events have been proposed to contribute to the major brain sequelae observed in premature infants with PVL, including injury to preOLs, axons, subplate neurons, migrating GABAergic neurons, and thalamus [16, 20, 38]. Primary injury in any one of these areas could lead to the OL cell death, hypomyelination and impaired cortical and thalamic development commonly observed in both the human brain following HI and our model of rodent brain injury [16]. It is well established, however, that developing neurons are highly dependent on trophic support for survival, and that target deprivation and failed tract formation also results in degeneration [8]. Delayed neurodegeneration in a systems-preferential manner is an important component to preterm brain injury, as it results in impairments in the human newborn that evolve into complex disabilities over time [8]. For example, cortical injury following HI will result in later thalamic damage due to loss of trophic support [8]. Detailed neuropathological examinations in human PVL have shown the thalamus to be similarly vulnerable, with neuronal loss, gliosis, and axonal degeneration present in 60% of cases [40]. Further, marked reductions in the density of layer V cortical neurons in human PVL cases have also been documented and may be reflective of injury secondary to necrosis in the underlying white matter [41]. These neuropathological findings in the human cortex corroborate our previous data indicating HI rat pups have significantly reduced cerebral mantle thickness [31]. Collectively, these data highlight the vulnerability of this region and are consistent with long-term MRI followup of older infants and children diagnosed with PVL as preterm babies that also demonstrate a reduction in the cerebral mantle, constituted by decreased cortical and white matter volume [23, 42]. Importantly in this investigation, we show that as injury to the periventricular white matter increases, the severity and frequency of cortical and subcortical grey matter neuronal degeneration increase with a regional predilection for the cortex, caudate putamen, and thalamus. We also document that all HI rat pups, even those without WM loss, exhibit a degree of axonal injury, as evidenced by presence of fractin immunoreactivity. During the peak period of vulnerability to PVL, cerebral white matter axons are rapidly growing. The occurrence of axonal injury in the necrotic foci of severe PVL has been known for years, but the widespread axonal degeneration in diffuse PVL, separate from focal necroses has only recently been documented [14, 38, 43–45]. Consistent with these observations, diffusion tensor imaging in noncystic PVL shows blunting of the normal maturational increase in fractional anisotropy in various axonal tracts [46–50]. Our data indicates that rats with moderate or severe loss of MBP have significantly increased axonal degeneration in the temporal-parietal cortex, caudate putamen, thalamus and internal capsule. As reported in prior animal studies, we also confirm that the hippocampus appears to be less susceptible to axonal injury and neuronal degeneration when exposed to HI at P6 and evaluated at P9 [51]. In our study hippocampal axonal injury occurs most notably, when WM injury is moderate to severe.

Subtle white matter and microstructural abnormalities in preterm infants are commonly associated with developmental impairment and abnormal visual, motor, and cognitive function [13, 18, 26]. Interestingly, we found that pups without evidence of gross periventricular white matter loss exhibited mild selective grey matter injury, as evidenced by mild axonal injury and neuronal degeneration, in the cortex, internal capsule, and caudate putamen; structures central to language processing and understanding, and motor and sensory function. Injury in these regions, even if mild, may be implicated in the neurocognitive disturbances noted in preterm survivors who do not demonstrate other clinical or radiological evidence of overt periventricular white matter injury [14]. Further, these findings demonstrate the increased necessity of combining traditional pathological techniques with high-resolution neuroimaging in animals. Just as diffuse white matter lesions were undetectable in preterm babies before the routine use of advance MRI sequences, subtle white matter abnormalities and HI changes to brain microstructure could go unrecognized in an animal model. Studies currently underway in our laboratory are addressing the connection between MBP loss, structural coherence of white matter, and the 3D course of axonal pathways following HI in the neonatal rodent.

In this investigation, all HI pups, including those without MBP loss, had significantly increased numbers of activated microglia and reactive astrocytes in white matter. Additionally, as white matter injury became increasingly severe, the numbers of activated microglia and reactive astrocytes increased in both white and grey matter, including the cortex, hippocampus, thalamus, and caudate putamen. Of note, when examining gliotic changes in the evaluated grey and white matter regions, a low to moderate level of baseline microglial activation was noted in the periventricular white matter and thalamus of control animals. Similarly, a low to moderate level of reactive astrocytes were also noted in the periventricular WM and hippocampus of controls. In the normal brain, microglia are first prominent in the forebrain in the 16–22 weeks of gestation and reach peak abundance in the cerebral white matter later in gestation [52–54]. In a recent longitudinal study of human brain, microglia density in white matter peaked during the greatest vulnerability to PVL (early third trimester), and then declined in white matter after 37 weeks gestation [16, 54]. Interestingly, as microglia declined in the white matter, their density increased in the cortex [16]. The presence of these cells in the uninjured brain is likely due to their function in a rapidly developing and dynamic brain, and these normal features are consistent with the recognized roles for microglia in brain development, including apoptosis, vascularization, axonal development, and myelination [20, 54]. The role of neuroinflammation in preterm brain injury has similarly been studied and microglia have been suggested to be a convergence point in the potentiation of HI and infection/inflammatory insults [16]. Premature infants are subject to numerous inflammatory conditions and microglia have been recognized as a prominent component of diffuse PVL [16, 55, 56]. Our findings related to the regional distribution of microglial activation, and astrogliosis confirm prior published reports of expression of these cellular subtypes in rodent models of HI induced brain injury [30, 57, 58]. The increase in reactive astrocytes and activated microglia in cortex, caudate, thalamus, and hippocampus may be a consequence of and/or a secondary pathophysiologic response to injury of the cortical neuronal populations, the oligodendrocyte precursors cells, and the subplate neurons that reside in regions adjacent to and in the periventricular WM region and cortical grey matter structures. Confirmatory of these mechanisms are previously published studies showing marked neuroprotection by agents such as doxycycline and minocycline that attenuate microglial activation and neuroinflammation [30, 57, 59].

## 5. Conclusion

In summary, the data presented here is the first to evaluate the relationship between degree of periventricular WM injury and its associated regional grey matter injury *in vivo* in a Long-Evans rat model of preterm HI brain injury. We show that as WM loss increases, the severity and frequency of cortical and subcortical grey matter injury increase with a regional predilection for the temporal-parietal cortex, internal capsule, caudate putamen, and thalamus. These findings are similar to injury patterns and susceptibilities noted in prior human preterm brain injury studies. Collectively, these data indicate that numerous cellular and molecular questions can be addressed in this translational Long-Evans rat model. This will allow for rapid progress in understanding the pathophysiology and appropriate avenues for intervention after HI injury in the developing nervous system.

## Figures and Tables

**Table 1:** Summary and comparison of study animal characteristics by hypoxia time.

Characteristics	Controls *n* = 10	1 hr 0 min *n* = 10	1 hr 5 min *n* = 9	1 hr 10 min *n* = 8	1 hr 15 min *n* = 11	1 hr 20 min *n* = 11
Postnatal day at study start/surgery	6	6	6	6	6	6
Postnatal day at sacrifice	9	9	9	9	9	9
Mean weight at PD 6, grams ± SEM	13.19 ± 0.45	13.61 ± 0.36	13.67 ± 0.32	14.08 ± 1.35	13.45 ± 0.67	13.19 ± 0.33
Mean weight at PD 9, grams ± SEM	19.53 ± 0.63	19.20 ± 0.46	19.70 ± 0.35	20.07 ± 0.34	19.78 ± 0.26	19.08 ± 0.40
Mean weight gain, grams ± SEM	6.34 ± 0.33	5.59 ± 0.27	6.03 ± 0.20	6.01 ± 0.21	6.33 ± 0.16	5.9 ± 0.20
Surgery time, min ± SEM	N/A	6.83 ± 1.38	9.33 ± 0.88	8.56 ± 0.73	10.18 ± 0.77	7.2 ± 0.64
Mean core temperature at surgery start, Celsius ± SEM	N/A	36.13 ± 0.40	35.2 ± 0.25	34.90 ± 0.18	34.90 ± 0.25	34.80 ± 0.40
Mean core temperature at surgery end, Celsius ± SEM	N/A	33.28 ± 0.62	31.9 ± 0.20	32.20 ± 0.32	32.20 ± 0.42	32.00 ± 0.28
Temperature decrease during surgery, Celsius ± SEM	N/A	2.9 ± 0.31	3.3 ± 0.25	3.0 ± 0.33	2.9 ± 0.56	2.8 ± 0.45

SEM: standard error of mean.

PD: postnatal day.

**Table 2:** Summary and comparison of study animal characteristics: by analysis group.

Characteristics	Controls *n* = 10	Grade 0 MBP Loss *n* = 16	Grade 1 MBP Loss *n* = 13	Grade 2 MBP Loss *n* = 9	Grade 3 MBP Loss *n* = 11
Postnatal day at study start/surgery	6	6	6	6	6
Postnatal day at sacrifice	9	9	9	9	9
Mean weight at PD 6, grams ± SEM	13.19 ± 0.45	13.56 ± 0.23	13.12 ± 0.28	13.76 ± 0.34	13.95 ± 0.32
Mean weight at PD 9, grams ± SEM	19.53 ± 0.63	19.68 ± 0.28	19.03 ± 0.34	19.67 ± 0.36	20.11 ± 0.32
Mean weight gain, grams ± SEM	6.34 ± 0.33	6.12 ± 0.13	5.91 ± 0.20	5.91 ± 0.21	6.16 ± 0.20
Surgery time, min ± SEM	N/A	8.06 ± 0.74	8.08 ± 0.94	9.56 ± 0.60	9.45 ± 0.86
Mean core temperature at surgery start, Celsius ± SEM	N/A	35.22 ± 0.31	35.21 ± 0.25	35.29 ± 0.21	35.16 ± 0.35
Mean core temperature at surgery end, Celsius ± SEM	N/A	32.77 ± 0.36	31.82 ± 0.45	31.99 ± 0.17	32.05 ± 0.24
Temperature decrease during surgery, Celsius ± SEM	N/A	2.45 ± 0.27	3.39 ± 0.54	3.36 ± 0.23	3.11 ± 0.36

SEM: standard error of mean.

PD: postnatal day.
